# Disk Evolution, Element Abundances and Cloud Properties of Young Gas Giant Planets

**DOI:** 10.3390/life4020142

**Published:** 2014-04-14

**Authors:** Christiane Helling, Peter Woitke, Paul B. Rimmer, Inga Kamp, Wing-Fai Thi, Rowin Meijerink

**Affiliations:** 1 SUPA, School of Physics & Astronomy, University of St Andrews, North Haugh, St Andrews KY16 9SS, UK; E-Mails: pw31@st-and.ac.uk (P.W.); pr33@st-and.ac.uk (P.B.R.); 2 Kapteyn Astronomical Institute, Postbus 800, Groningen 9747 AV, The Netherlands; E-Mails: kamp@astro.rug.nl (I.K.); meijerink@astro.rug.nl (R.M.); 3 Laboratoire d’Astrophisique de Grenoble, CNRS/Université Joseph Fourier (UMR5571) BP 53, Grenoble cedex 9 F-38041, France; E-Mail: wing-fai.thi@obs.ujf-grenoble.fr; 4 Leiden Observatory, Leiden University, P. O. Box 9513, Leiden 2300 RA, The Netherlands

**Keywords:** astrochemistry, element abundances, extra-solar planets, gas giants, planet formation, cloud formation

## Abstract

We discuss the chemical pre-conditions for planet formation, in terms of gas and ice abundances in a protoplanetary disk, as function of time and position, and the resulting chemical composition and cloud properties in the atmosphere when young gas giant planets form, in particular discussing the effects of unusual, non-solar carbon and oxygen abundances. Large deviations between the abundances of the host star and its gas giants seem likely to occur if the planet formation follows the core-accretion scenario. These deviations stem from the separate evolution of gas and dust in the disk, where the dust forms the planet cores, followed by the final run-away accretion of the left-over gas. This gas will contain only traces of elements like C, N and O, because those elements have frozen out as ices. ProDiMo protoplanetary disk models are used to predict the chemical evolution of gas and ice in the midplane. We find that cosmic rays play a crucial role in slowly un-blocking the CO, where the liberated oxygen forms water, which then freezes out quickly. Therefore, the C/O ratio in the gas phase is found to gradually increase with time, in a region bracketed by the water and CO ice-lines. In this regions, C/O is found to approach unity after about 5 Myrs, scaling with the cosmic ray ionization rate assumed. We then explore how the atmospheric chemistry and cloud properties in young gas giants are affected when the non-solar C/O ratios predicted by the disk models are assumed. The Drift cloud formation model is applied to study the formation of atmospheric clouds under the influence of varying premordial element abundances and its feedback onto the local gas. We demonstrate that element depletion by cloud formation plays a crucial role in converting an oxygen-rich atmosphere gas into carbon-rich gas when non-solar, premordial element abundances are considered as suggested by disk models.

## 1. Introduction

Element abundances are critical parameters to predict the atmospheric composition of exoplanets and to understand their formation and evolution, including potentially the emergence of life. Extrasolar gas giants are commonly assumed to have elemental abundances similar to those of their host stars. These stars themselves can be reasonably well measured, for example (in case of the Sun) by high-resolution spectroscopy in combination with time-dependent numerical simulations of the photosphere, meteorite studies, or astroseismology [1]. However, when considering the process of planet formation in a protoplanetary disk, which involves a segregation of gas, dust and ice phases, the assumption that the element mix of the host star must be the same as in the gas giants’ atmospheres becomes questionable. This has far-reaching consequences for the spectroscopic analysis of planetary spectra, including the search for bio-signatures [2,3,4]. Following the standard core-accretion model of planet formation [5,6], the refractory elements are initially present mostly in form of μm-sized dust particles, which undergo a complex evolution eventually leading to km-sized planetesimals. The planetesimals are gravitationally attracted to each other, and collide to form larger parental bodies that later become planetary cores [7,8].

At the end of the evolution from dust to planet cores, the planet feeding zone is expected to be mostly devoid of smaller dust particles [9], and the remaining gas in the planet feeding zone is expected to contain only minor traces of refractory elements. Elements which are able to form ices on the surface of the refractory grains in protoplanetary disks, in particular oxygen, carbon and nitrogen, will also be depleted to an extent, depending on local temperature, though less than the refractory elements. The ices play an essential role in the dust growth process as “glue” or “cement” during planet formation [10]. The dust particles have been in contact with the gas in the disk for *>* 10^6^ years, which is certainly long enough to cause most of the gaseous oxygen in the disk midplane to form H_2_O ice outside the water “ice-line” and for most of the gaseous carbon to form CO ice outside the CO “ice-line” [11]. The elements bound in those ices should then rather follow the dust than the gas dynamical evolution. Only at the very end of the planet formation process, the overwhelming majority of the mass of the gas giant will be accreted onto the proto-planet in a rapid run-away phase [12], using up the remaining gas in the planet feeding zone and possibly forming a gap. The timescale for gas accretion onto the proto-planet is about two orders of magnitude shorter than the growth timescale of the solid core [13]. At this late stage, the gas should contain only traces of refractory elements, and possibly also very little amounts of the “ice elements” O, C and N, depending on local temperature, *i.e*., position in the disk. The resulting planetary atmosphere will hence be extremely metal-poor in the first place. Late bombardment with left-over planetesimals [14,15] will cause an element re-enrichment leading once more to a change of the atmospheric composition. The opacity of the atmosphere surrounding the planetary core plays an important role for the critical mass that inhibits further accretion [16].

Thus, the formation of gas giants via core-accretion means that, first, the ice and volatile elements segregate. Then the gas and icy dust evolves separately. Finally, the icy dust and the gas combine in a specific order, forming a planet. It would be a strange coincidence if all these complicated processes would result in gas giant surface element abundances that resemble those of their host stars. We should rather expect a large variety of the atmospheric element abundances of gas giants, depending on when and where the planets form.

In this paper, we first study the segregation and evolution of gas and ice in protoplanetary disk models to predict the element abundances of the gas that will finally be accreted onto the proto-planets (Section 2). We show that the resulting carbon-to oxygen ratio (C/O) is expected to be larger than the primordial value, and increase further with time, in particular between the water snowline (≈150 K) and the CO ice-line (≈20 K), where mostly water freezes out. Similar results have been recently obtained in [17], who schematically discussed the relative segregation of carbon, oxygen, and nitrogen in the disk, causing the C/O and C/H ratios to differ significantly from those of the host stars. Gaseous C/O ratios close to unity between the water and CO ice-lines, mainly driven by the formation of water, CO and CO_2_ ices [17]. The time-dependent ice composition in the midplane of T Tauri disks has been studied by [11,18], who found agreement with measured chemical compositions of comets in their model, after long integration times (10 Myrs) at 10 AU, using a relatively high cosmic ray ionization rate of 5 × 10^−17^ s^−1^ and a sophisticated treatment of the secondary cosmic ray induced photo reactions, the rates of which are enhanced due to an increased ratio of UV gas absorption with respect to dust absorption, driven by dust growth with respect to interstellar conditions.

We expect the metal-poor gas in protoplanetary disks to lead to unusual element abundances in planets, in particular the element abundances in gas giant atmospheres, although the dynamical details of the actual planet formation process need further investigation [16]. Tentative detections of carbon-rich planets have been announced, concerning the planets WASP 12b and 55 Cancri e, respectively [19,20]. However, 65 orbits of WFC3-IR grism observations could not find any evidence for C*/*O *>* 1 in the atmosphere of WASP 12b [21], as was reported [19]. And in the case of 55 Cancri e, is was shown that the abundance analysis of the host star 55 Cancri [22] (used in [20]) was probably erroneous due to a unsuitable choice of a zero-excitation oxygen line [23].

Most of the presently known extrasolar planets orbit somewhat metal-rich host stars [24]. However, this simple relation holds only for giant gas planets, but not for Neptune-sized planets. It was argued that the Sun has a depletion of refractory to volatile elements of about 20% with respect to planet-free solar twins [25]. This abundance deficit roughly matches the mass of the terrestrial planets. One possible explanation for these deficiencies is to assume an early formation of ∼10 km bodies, which later formed the terrestrial planets before the majority of the Sun’s mass (excluding the ∼10 km bodies) was accreted from the proto-solar disk. The abundance peculiarities can also be found in solar-like stars that are known to have close-in giant planets [25].

Our knowledge about the chemical composition of exoplanet atmospheres is prompted by transit observations of close-in planets, e.g., [26,27,28], or by bulk properties like global density estimates, e.g., [29]. Recent observation of the four directly imaged HD 8788 planets, however, suggest spectral diversity amongst co-eval objects of similar luminosity. The authors report on tentative detections of CH_4_, C_2_H_2_, CO_2_ and HCN [30]. A more complete understanding of exoplanet atmospheres hinges on the detailed atmosphere modeling that must include cloud formation, photochemistry and global circulation. The element abundances are essential parameters to all those models, and a simple scaling of a metallicity parameter, e.g., from [26,31], is far from realistic, as we will demonstrate in Section 3 of this paper. Planetary atmosphere chemistry does not only depend on the initial element abundances but also on the cloud formation process that depletes condensable elements and hence determines the remaining gas composition and radiative cooling processes. Cloud formation will impact all elements involved (Fe, Ti, Al, O, . . . [2,3]) and thereby change the C/O-ratio in such atmospheres (see Figure 2 and Figure 3 in [4]). Cloud layers have a large impact on the atmospheric structure and the spectral appearance of ultra-cool low-mass objects, like brown dwarfs and planets. Thus, a direct abundance analysis of exoplanets like WASP 12b, possibly with future instruments like JWST, must take these effects into account carefully. Different cloud models make different predictions for the remaining gas-phase abundances resulting in different molecular abundances [32]. Clouds are expected to occur in a variety of physical phases and chemical compositions, depending on temperature and pressure, from cold icy hazes, over liquid droplets, to hot solid gemstones. Beside temperature and pressure, the cloud formation process is controlled by the elemental composition of the atmospheric gas, which in turn is drastically reduced by the consumption of condensable elements into cloud particles and subsequent rain-out [2].

Section 2 summarizes the disk chemistry model that we use to predict gas and ice elemental abundances in proto-planetary disks. Section 3 introduces our model for planetary atmospheres and cloud formation. Inspired by the results of the disk models, we consider unusual element abundances, in particular large ratios C*/*O ≲ 1 in young gas giant atmospheres. In Section 4, we demonstrate that such non-standard oxygen abundances have a strong impact on the atmospheric structure and cloud properties in the atmosphere, like the cloud base, cloud particle number densities, and mean grain sizes. We further show that condensation of oxygen-rich dust may cause the C/O ratio to tip over locally, C*/*O*>*1, and we show that the abundances of astrobiologically interesting molecules like H_2_O, CH_4_, NH_3_, C_2_H_2_, C_2_H_6_ may increase near the cloud top.

## 2. Gas and Ice Abundances in Protoplanetary Disks

In order to model the chemical composition of the gas and ice in the disk as function of time, we follow a two-stage modeling strategy. In *disk modeling stage 1*, we simulate the chemistry in the dark cores of molecular clouds by advancing our chemical rate network under the corresponding temperature, density, and radiation field conditions for an assumed lifetime of the dark core. The resulting concentrations are then taken as initial conditions for the disk simulations in *disk modeling stage 2*. This is a very much simplified approach which ignores the complicated hydrodynamical star and disk formation processes itself, as well as the changing conditions in the disk during class-0 and class-I, although these phases are relatively short. Instead, we reset our clock at the beginning of stage 2, and then advance our chemistry further under the local temperature, density, dust, and radiation field conditions in the disk.

More sophisticated models for the early disk phases, which evolve the chemistry along streamlines from hydro-models for star and disk formation, have been carried out by [33]. In this paper, we are interested in the long-term evolution of the chemistry, and hence concentrate on class-II disks. Protoplanetary disks are observed to survive for about 3–10 Myrs before they disperse [34,35]. We evolve the disk chemistry for 10 Myrs, and even beyond, to study the asymptotic behavior towards kinetic chemical equilibrium which provides an interesting special case, providing additional understanding of the complete chemical paths for disk midplane evolution.

For our chemical simulations, we apply the radiation thermo-chemical disk code ProDiMo [36]. The 2D code consistently solves the dust continuum radiative transfer, with the dust component in radiative equilibrium, the gas heating and cooling balance (70 heating processes, 64 cooling processes), the gas phase and ice chemistry, and the non-LTE line transfer in class-II protoplanetary disks, with recent updates described by [37,38]. The chemical model consists of 12 elements (H, He, C, N, O, Mg, Si, S, Fe, Ne, Ar, PAH) and 166 species, including vibrationally excited H_2_, 30 ice species with adsorption energies assumed as listed in Table 1, and 5 charging states of PAHs. Concerning the reaction rates, we have experimented with two networks: the UMIST-2012 rates [39] and the OSU-2010 rates with 2012 erratum [40,41]. In both cases, we select all reactions among our selected species, and add the same set of additional reactions for vibrationally excited H_2_, UV ionization and photo-dissociation (based on detailed UV cross sections from the Leiden database [42], coupled to the radiative transfer), X-ray reactions [43], PAH charging reactions, and ice formation (adsorption, thermal desorption, UV desorption, cosmic ray desorption), as well as H_2_ formation on grains and some surface chemistry, see [38].

**Table 1:** Assumed adsorption energies for some important ices, after [39].

**Ice species**	**O**	**OH**	**H_2_O**	**O_2_**	**CO**	**CO_2_**	**CH_3_CO**	**C_2_H_2_**	**CH_3_**	**CH_4_**	**N**	**N_2_**	**NH_3_**
*E*_ads_ [K]	800	2850	4800	1000	1150	2990	4930	1400	1175	1090	800	790	5534

### 2.1. Stage 1: The Dense Core Simulations

Stage 1 of our simulation is a simple one-point model for the dense cores of molecular clouds, where we advance our chemical rate network for a certain time, from initial atomic abundances typical for the diffuse interstellar medium.

For the dense core conditions we adopt the following values as recommended for TMC-1 by [39]: temperatures *T*_gas_ = *T*_dust_ = 10 K, density *n*_〈H〉_ = 10^4^ cm^−3^, extinction *A_V_* = 10, dust particle density *n*_dust_ = 1.8 × 10^−8^ cm^−3^ and dust size *a* = 0.1 μm. We assume large gas column densities in order to switch off the X-rays and have large molecular shielding factors. The integration time is chosen to be 1.7 × 10^5^ years, the assumed lifetime of TMC-1 according to [39]. We note that these parameters are debated in the literature, see e.g., [44,45], where the resulting abundance of O_2_ is crucial, because O_2_ is not detected (observed concentration is *<*8×10^−8^ in TMC-1 [39]). Very recently, however, [46] reported on a 4.5*σ* detection of O_2_ from the molecular cloud surrounding the deeply embedded low-mass class-0 protostar NGC 1333-IRAS 4A. For the initial atomic abundances, we adopt the values from [39], see their Table 3.

**Table 2:** Assumed atomic and calculated abundances for dense core conditions. The latter are taken as initial values for the disk simulations in modeling stage 2. Numbers are particle concentrations with respect to hydrogen nuclei, “#” denote ice species, notation *x*(−*y*) means *x* × 10^−*y*^. We only list a few species here, that are either abundant in the initial atomic (column “atomic”) or in the resultant chemical state.

	**Atomic**	**UMIST 2012 ^⋆^**	**OSU 2010 ^⋆^**
H	5 (−5)	2.6 (−4)	2.2 (−4)
H_2_	0.5	0.5	0.5

He	0.09	0.09	0.09

C^+^	1.4 (−4)	3.0 (−8)	1.5 (−8)
CO	0	5.9 (−5)	5.1 (−5)
C	0	4.1 (−5)	4.0 (−5)
C#	0	2.2 (−5)	2.3 (−5)
CO#	0	1.1 (−5)	8.1 (−6)

N	7.5 (−5)	4.9 (−5)	4.3 (−5)
N#	0	1.7 (−5)	1.6 (−5)
N_2_	0	3.9 (−6)	7.0 (−6)
N_2_#	0	5.8 (−7)	8.9 (−7)
HCN	0	1.1 (−7)	1.4 (−7)
HNC	0	1.0 (−7)	1.2 (−7)

O	3.2 (−4)	1.8 (−4)	1.9 (−4)
O#	0	4.1 (−5)	4.6 (−6)
OH#	0	1.9 (−5)	1.8 (−5)
H_2_O#	0	1.1 (−5)	7.6 (−6)
H_2_O	0	3.0 (−7)	7.6 (−7)
O_2_	0	1.3 (−8)	1.2 (−8)

S^+^	8 (−8)	7.4 (−10)	2.4 (−9)
S	0	6.1 (−8)	5.9 (−8)
S#	0	1.6 (−8)	1.2 (−8)
CS	0	2.0 (−9)	4.8 (−9)
CS#	0	6.1 (−10)	1.9 (−9)

Si^+^	8 (−9)	1.0 (−9)	7.6 (−11)
Si	0	3.9 (−9)	5.7 (−9)
SiO	0	1.9 (−9)	6.6 (−10)
Si#	0	8.9 (−10)	1.5 (−9)
SiO#	0	3.3 (−10)	1.3 (−10)

Mg^+^	7 (−9)	5.2 (−9)	4.9 (−9)
Mg	0	1.5 (−9)	1.7 (−9)
Mg#	0	3.6 (−10)	4.1 (−10)

Fe^+^	3 (−9)	2.3 (−9)	2.2 (−9)
Fe	0	5.9 (−10)	7.5 (−10)
Fe#	0	7.8 (−11)	8.3 (−11)

Ne	6.9 (−5)	6.9 (−5)	6.9 (−5)

Ar	1.5 (−6)	1.5 (−6)	1.5 (−6)

PAH	2.8 (−9)	8.1 (−10)	6.4 (−10)
PAH*^−^*	0	2.0 (−9)	2.1 (−9)

^⋆^ Reactions among our selection of species, and combined with other reactions, see text.

The results of the dark core simulations are summarized in Table 2. We get an oxygen-rich gas where oxygen and nitrogen are mostly present in form of neutral atoms, and carbon in form of CO, with smaller quantities already frozen out as O#, OH#, H_2_O#, C#, CO#, N# and N_2_# ices. The low density and short lifetime assumed for TMC-1 avoid large concentrations of O_2_, and favor the formation of simple (e.g., atomic) ices, which have abundant atomic/molecular counterparts in the gas phase, according to our simple ice chemistry. However, it must be noted that if we integrated our reaction network for slightly longer times, or higher densities, the O_2_ concentration would increase rapidly, as is true in the original, pure gas-phase, UMIST-2012 network see [39]. Adding all gaseous concentrations together,

(1) $$\langle {\text{O}}_{\text{gas}}\rangle =\sum_{i\;(\text{only gas})}s_{\text{O}}^{i}\frac{n_{i}}{n_{\langle \text{H}\rangle }}$$

where, for example, $s_{\text{O}}^{i}$ is the stoichiometric coefficient of oxygen in molecule *i*, we get *∊*_O_ = 12 + log_10_〈O_gas_〉 = 8.380 (8.381), *∊*_C_ = 8.030 (8.037), and *∊*_N_ = 7.754 (7.755), where the numbers in brackets refer to the model with the OSU rates. These values are close to the atomic abundances assumed in the first place, *∊*_O_ = 8.505, *∊*_C_ = 8.146 and *∊*_N_ = 7.875.

### 2.2. Stage 2: The Disk Simulations

We consider an example class-II protoplanetary disk with parameters as listed in Table 3. The parameters have been carefully chosen to match various continuum and line observations of class-II T Tauri stars concerning SED shape (clearly visible 10 μm and 20 μm silicate emission features, decreasing SED-slope beyond 20 μm, typical for non-transitional disks), near-IR excess (0.15 L_⊙_ between 2 μm and 7 μm), mm-flux (130 mJy at 140 pc), mm-slope *β* = −Δ log(*F_ν_*)*/*Δlog(λ) − 2 = 0.3, [OI] 63 μm line flux (4 × 10^−17^ W*/*m^2^ at 140 pc) and various CO sub-mm line fluxes. The stellar parameters describe a T Tauri star of spectral type K7 with an age of about 1.6 Myrs. The disk extends radially from 0.07 AU to 200 AU, and has an assumed radial surface density profile as $\Sigma (r)\propto r^{-∊}\text{exp}\left(-\frac{r}{R_{\text{tap}}}\right)$. The resulting densities in the midplane are *n* ≈ (10^16^ *−* 10^6^) cm^−3^, depending on *r*, and the midplane temperatures are as low as *T* ≈ (300 *−* 5) K, encompassing radii *r* =0.15*−*200 AU (disregarding the hot inner rim with temperature ≈ 1500 K here). Dust settling is included according to [47], assuming an equilibrium between upward turbulent mixing and downward gravitational settling, which leads to higher dust/gas ratios in the midplane, in particular in the outer midplane. The midplane regions are entirely shielded from the stellar UV and even from the stellar X-rays. The vertical extinction of the midplane is *A_V_* ≈ 1500 at 1 AU and still *A_V_* ≈ 15 at *r* = 50 AU. The radial *A_V_* are much larger.

The above described conditions are typical for the midplane only, and we will entirely focus on the central midplane results in this paper, where planet formation occurs. Since stellar UV and X-rays can penetrate the upper, thinner disk layers, these layers have a very different chemistry. The upper layers are much warmer, and the high-energy photons drive an active X-ray/photo-chemistry producing most of the observable line emissions. In these layers, the chemical relaxation timescales are short, and the application of kinetic chemical equilibrium is justified [36]. Therefore, it would be an error to generalize the chemical midplane results as described in this paper to the entire disk, or to the line-emitting regions.

We use the dense core concentrations from Table 2 as initial values, reset the clock, and integrate forward our chemical rate network in disk configuration, using a well-iterated spatial density, temperature and radiation field structure of the disk obtained before with a standard model, where kinetic chemical equilibrium is assumed. The physical conditions in the 2D disk are hence kept constant in time during modeling stage 2.

**Table 3:** Parameters of the T Tauri type, class-II protoplanetary disk model.

**Quantity**	**Symbol**	**Value**
stellar mass	*M*_⋆_	0.7 *M*_⊙_
stellar luminosity	*L*_⋆_	1.0 *L*_⊙_
effective temperature	*T*_eff_	4000 K
UV luminosity	*L*_UV_	0.01 *L*_⊙_
X-ray luminosity	*L*_X_	10^30^ erg/s

minimum dust particle radius	*a*_min_	0.05 μm
maximum dust particle radius	*a*_max_	3 mm
dust size dist. power index	*a*_pow_	3.5
dust settling turbulence parameter	*α*	0.001
max. hollow-sphere volume ratio	*V*_max_*_,_*_HS_	0.8
dust composition	Mg_0.7_Fe_0.3_SiO_3_	60%
(volume fractions)	amorph. carbon	15%
	vacuum	25%

disk gas mass	*M*_gas_	3 × 10^−2^ *M*_⊙_
disk dust mass	*M*_dust_	3 × 10^−4^ *M*_⊙_
inner disk radius	*R*_in_	0.07 AU
tapering-off radius	*R*_tap_	50 AU
outer disk radius	*R*_out_	200 AU
column density power index	*∊*	1.0
reference scale height	*H*_0_	10 AU
reference radius	*r*_0_	100 AU
flaring power index	*β*	1.12

cosmic ray ionization rate	*ζ*_CR_	1.3 × 10^−17^ s^−1 (⋆)^
PAH abundance rel. to ISM	*f*_PAH_	0.01
chemical heating efficiency	*γ*^chem^	0.2

(⋆) Standard value according to [39,40].

### 2.3. Results

**Figure 1:**
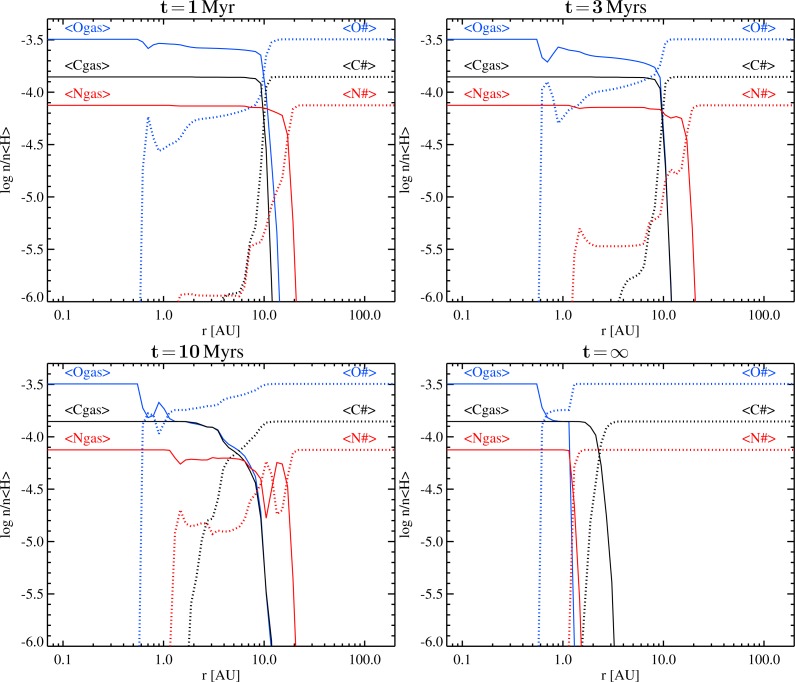
Time evolution of total gas (solid) and total ice (dotted) abundances of oxygen (blue), carbon (black), and nitrogen (red) in the midplane, according to a time-dependent ProDiMo model, based on the UMIST-2012 rates, for a T Tauri type protoplanetary disk. The y-axis shows the concentration with respect to hydrogen nuclei, take this value +12 to get the usual element abundances *∊ (i.e., ∊_H_* =12).

**Figure 2:**
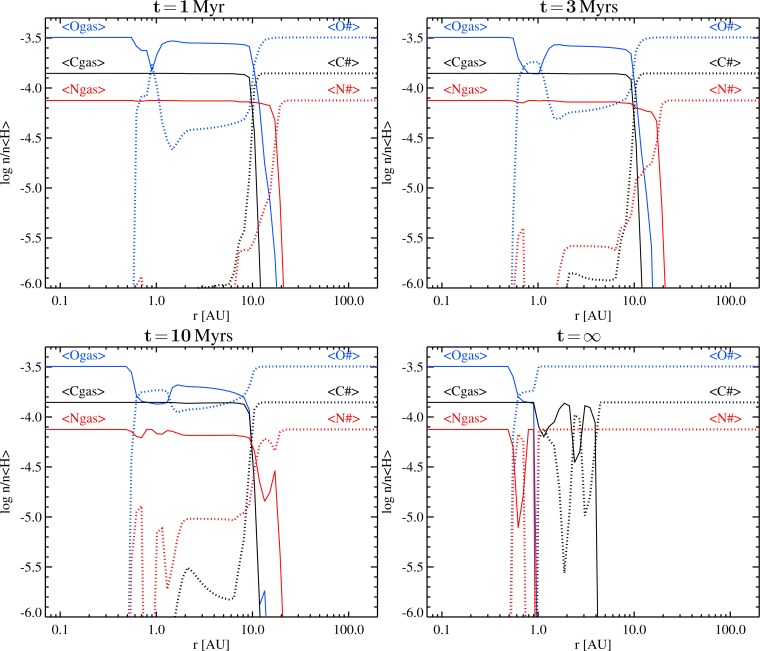
Same as Figure 1, but for the ProDiMo model based on the OSU-2010 rates.

The time-dependent segregation of carbon, nitrogen and oxygen into gas and ice in the midplane is shown in Figure 1 and Figure 2, based on the models with the UMIST-2012 and the OSU-2010 rates, respectively. According to the model, the midplane is chemically subdivided into three different radial zones, separated by the H_2_O and CO ice-lines. The innermost zone (*r <* 0.6 AU, *T <* 150 K, *n*_〈H〉_ *>* 5 × 10^14^ cm^−3^) is too hot for any stable ices, hence the gas abundances are equal to the assumed total element abundances. The sum of gas and ice abundances is prescribed by the “element abundances” in the model. Abundant molecules here are H_2_O, CO, CO_2_, HCN, HNC, CH_4_ and NH_3_, similar to the ultra-cool atmospheres of brown dwarfs or giant gas planets (compare Figure 10).

In the outer zone, beyond the CO ice-line (*r >* 13 AU, *T <* 25 K, *n*_〈H〉_ *<* 5 × 10^11^ cm^−3^), oxygen and carbon are quickly converted to ices (mainly H_2_O# and CO#). Nitrogen freezes out in form of N# and N_2_# a bit further out (*r >* 20 AU), because of the slightly lower adsorption energies. These statements are valid already after some 100 years. The initial freeze-out of condensable molecules is actually a very fast process (see Table 4). The adsorption timescale *τ*_ads_, *i.e*., the timescale for a molecule to hit and stick to the surface of a grain is

(2) $$\tau_{\text{ads}}^{-1}=\alpha n_{\text{dust}}4\pi \langle a^{2}\rangle v_{\text{th}}$$

where *α* ≈ 1 is the sticking coefficient, *n*_dust_ [cm^−3^] the local dust particle density, 〈*a*^2^〉 the mean of the squared dust particle radii, averaged over the size distribution, and $v_{\text{th}}=\sqrt{kT/(2\pi m)}$ the thermal velocity of a molecule with mass *m*. In the disk model, for a molecule like water (*m* = 18 amu), this timescale is as short as 1 sec at the inner rim, and 100 years at the outer radius, despite the tapering-off surface density assumed. What turns the ice formation actually into a slow process is the chemical conversion of the gas phase into condensable molecules prior to freeze-out.

Thus, already after 1 Myrs, the distant midplane gas contains practically no molecules other than H_2_. The atmosphere of a Uranus or Neptune-like planets, if composed of such gas alone, would contain practically no oxygen, carbon or nitrogen.

In the sandwich zone between the H_2_O and the CO ice-lines, that is between 0.6 AU and 13 AU in the particular model, the results are time-dependent. The earlier epochs (*t* ≲ 3 Myrs) are characterized by the slow build up of additional H_2_O# from the abundant gas phase molecules CO, CO_2_, O_2_, N and N_2_. The slow formation of water ice is what makes our results differ from [17]. This is because of the initial formation of O_2_ parallel to H_2_O, and the temperatures being too warm for O_2_ to freeze out. This way, most of the gaseous oxygen in the midplane is soon locked into the stable and chemically inert O_2_. In order to form additional H_2_O# under those circumstances, the O_2_ must be dissociated first, and this proceeds in the model either by cosmic ray ionizations or by reactions with C^+^, which are both slow processes.

**Figure 3:**
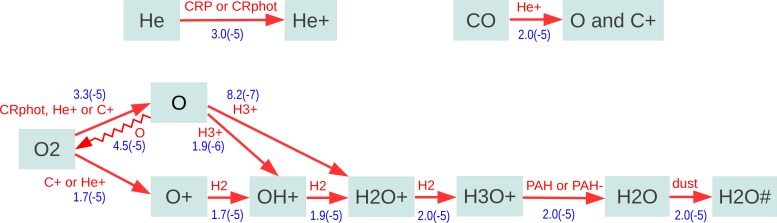
Destruction of molecular oxygen and formation of water ice, after 1 Myr in the midplane of the UMIST-2012 model at 2.5 AU, where temperature and density are 75 K and 3 × 10^13^ cm^−3^, respectively. “#” denotes ice species. The red species indicate the reaction partners, and the blue numbers are the reaction rates in s^−1^cm^−3^.

Figure 3 shows some details of the chemical paths involved. Initially, a helium atom is ionized by cosmic rays, and the He^+^ collides with a CO molecule to dissociate it into C^+^ and O. The C^+^ then attacks O_2_. If O_2_ is dissociated into neutral O atoms, quick radiative association reactions with other O atoms will re-form O_2_. However, when O^+^ is produced, there is a quick linear reaction chain which stepwise adds hydrogen atoms via reactions with H_2_ to form H_3_O^+^, and H_3_O^+^ then recombines at the surface of PAH molecules to form H_2_O, which then freezes out. Simplistically speaking, for every CO molecule un-blocked by cosmic rays, there will be one O_2_ molecule destroyed, and one new H_2_O# ice unit formed. The effective destruction timescale for O_2_, in the example shown, is

(3) $$\tau_{{\text{O}}_{2}}=\frac{n_{{\text{O}}_{2}}}{dn_{{\text{O}}_{2}}/\mathrm{dt}}=\frac{1.6\times 10^{9}{\text{cm}}^{-3}}{2.0\times 10^{-5}{\text{s}}^{-1}{\text{cm}}^{-3}}\approx 2.5\;\text{Myrs}$$

in the UMIST-2012 model, and ≈ 6 Myrs in the OSU-2010 model. The difference between the two models can be traced back to the CR induced secondary UV reaction He + CRphot → He^+^ + e*^−^*, in addition to the primary reaction He + CRP → He^+^ + e*^−^*. The secondary reaction, which about doubles the ionization rate of He, seems new in UMIST-2012 (compared to UMIST-2006), and was apparently not incorporated into the OSU-2010 reaction rates either. Consequently, our OSU-2010 model has about a factor of two less He^+^, and accordingly less C^+^, both required to form ionized oxygen, the main precursor of water.

As O_2_ is slowly consumed and converted into H_2_O#, the gaseous C/O-ratio increases and reaches unity after about 5 Myrs, but then C/O does *not* increase further. All timescales mentioned in the remainder of the text belong to the UMIST-2012 model, and scale with the assumed cosmic ray ionization rate. Although CO is continuously dissociated by cosmic rays (timescale about ∼ 6 Myrs in the UMIST-2012 model, ∼ 14 Myrs in the OSU-2010 model), the liberated O rather reacts with other molecules like CS and CN to reform CO. Some tiny amounts of H_2_O# do actually form via OH, but the associated timescale to convert CO into H_2_O#, in case C*/*O≈1, is huge, larger than 100 Myrs.

**Table 4:** Carbon, nitrogen and oxygen gas abundances at selected times and locations in the disk, according to disk models using the UMIST-2012 and OSU-2010 chemical rates.

	**UMIST-2012**					**OSU-2010**				
	**0.3 AU**	**1 AU**	**3 AU**	**10 AU**	**30 AU**	**0.3 AU**	**1 AU**	**3 AU**	**10 AU**	**30 AU**
*t*=0										

*∊*(O)	8.38	8.38	8.38	8.38	8.38	8.38	8.38	8.38	8.38	8.38
*∊*(C)	8.03	8.03	8.03	8.03	8.03	8.04	8.04	8.04	8.04	8.04
*∊*(N)	7.75	7.75	7.75	7.75	7.75	7.75	7.75	7.75	7.75	7.75

*t*=100 years										

*∊*(O)	8.51	8.49	8.46	8.35	2.41	8.51	8.49	8.47	8.36	4.47
*∊*(C)	8.15	8.15	8.14	7.83	5.56	8.15	8.15	8.14	7.84	4.63
*∊*(N)	7.88	7.87	7.87	7.86	5.03	7.88	7.88	7.87	7.87	2.55

*t*=1 Myr										

*∊*(O)	8.51	8.46	8.42	8.12	1.37	8.51	8.32	8.45	8.26	1.43
*∊*(C)	8.15	8.15	8.14	7.70	1.24	8.15	8.15	8.14	7.76	1.33
*∊*(N)	7.88	7.88	7.87	7.85	1.75	7.88	7.88	7.87	7.86	1.95

*t*=3 Myrs										

*∊*(O)	8.51	8.42	8.33	7.65	0.24	8.51	8.14	8.42	8.08	0.37
*∊*(C)	8.15	8.15	8.14	7.57	0.83	8.15	8.15	8.14	7.68	0.93
*∊*(N)	7.88	7.88	7.85	7.80	1.77	7.88	7.87	7.86	7.82	2.04

*t*=10 Myrs										

*∊*(O)	8.51	8.27	8.09	6.80	−1.53	8.51	8.13	8.30	7.61	−1.53
*∊*(C)	8.15	8.15	8.09	6.79	−0.25	8.15	8.15	8.14	7.60	−0.12
*∊*(N)	7.88	7.87	7.80	7.37	1.73	7.88	7.84	7.82	7.71	2.02

*t*=30 Myrs										

*∊*(O)	8.51	8.15	7.29	1.83	−1.53	8.51	8.13	8.06	7.18	−1.54
*∊*(C)	8.15	8.15	7.30	2.09	−0.39	8.15	8.15	8.06	7.19	−0.26
*∊*(N)	7.88	7.87	6.78	1.64	1.68	7.88	7.83	7.61	6.82	1.96

*t*=∞										

*∊*(O)	8.51	8.15	−7.62	−24.8	−13.8	8.51	5.12	−5.18	−24.8	−13.8
*∊*(C)	8.15	8.15	6.73	−3.23	−2.38	8.15	7.93	8.03	−2.95	−2.02
*∊*(N)	7.88	7.87	−4.92	−29.3	−14.6	7.88	1.92	−12.9	−29.6	−14.5

At later epochs (*t* ≳ 10 Myrs) additional, very stable ices with rare gaseous counterparts are formed, in particular NH_2_#, NH_3_#, C_2_H_2_#, CH_3_OH#, CH_3_# and CH_4_#. In fact, the simple ices formed in the first place are now slowly converted into these “late ices” (see also [11,18]). Since our model has only very limited surface chemistry [38], this conversion requires to temporarily evaporate the simple ices (by cosmic ray desorption), to convert the respective molecules in the gas phase into different ones by cosmic-ray chemistry, and then to freeze out the new molecules. These processes are extremely slow.

At an age of about 10 Myrs, considerable fractions of the late ices have built up, and the sandwich zone starts to shrink from the outside in. The outer part of the former sandwich zone joins the outer disk in becoming virtually molecule-free (except for H_2_), while the simple ices are steadily converted into their most stable, complex forms. The lower right plots in Figure 1 and Figure 2 shows the “fictive end stadium”, calculated in kinetic chemical equilibrium, where nitrogen disappears from the gas phase at about 1.5 AU, triggered by NH_3_ condensation, and gaseous carbon disappears at *r* ≳ 3 AU, due to the condensation of C_2_H_2_ and CH_3_OH ices. The chemical equilibrium model is also featured by C*/*O≫1 outside of about 1.5 AU where the remaining gas is devoid of CO. Instead, organic molecules are abundant, in form of small hydro-carbon chain molecules like C_2_, C_3_, C_3_H, C_2_H_2_ and C_3_H_2_.

Because all chemical processes described above are driven by cosmic ray ionization, the associated timescales are density-independent, *i.e*., the whole disk will undergo the chemical conversion C*/*O → 1 in a coherent way, between the H_2_O and CO ice-lines. However, the initial formation of the stable gases CO, O_2_, CO_2_, N_2_, etc., depends on local conditions and that explains the differences at one particular time in Figure 1 and Figure 2. Thus, cosmic rays provide a “clock” for the chemical conversion C*/*O → 1, driven by water ice formation, in the middle sections of protoplanetary disk midplanes (see Table 4).

### 2.4. Discussion

In this paper, we have investigated the chemical pre-conditions for planet formation, in terms of gas and ice abundances as function of time and position in the midplane of a protoplanetary disk. Under the dense and shielded conditions, the chemical composition in the disk midplane soon becomes quite simple. Only the most stable, almost inert, neutral molecules like H_2_O, O_2_, CO, CO_2_, CH_4_, N_2_ and NH_3_ will soon contain the vast majority of the elements, similar as in brown dwarf atmospheres. At radii where the disk midplane is cold enough for the corresponding ice phases to be thermally stable, those molecules freeze out after short times (≪ 10^3^ year). After that initial short period of relaxation, the chemistry then “comes to a halt”, meaning that almost no chemical processes occur anymore in the disk midplane – the chemical timescales increase towards millions of years.

All remaining chemical activity is then entirely due to cosmic ray (CR) hits, and we have applied a standard CR ionization rate of H_2_ of 1.3×10^−17^ s^−1^ [39] throughout the disk, which provides a slowly ticking clock, as with every dissociated O_2_ and CO molecule, there are opportunities to form other molecules which can freeze out, like water. The selective freeze-out of molecules containing oxygen leads to C*/*O → 1 in the gas phase inward of the CO ice-line (≈ 20 K), on timescales of several Myrs. The question arises what happens if cosmic rays do not even reach the disk, but are shielded by magnetic fields or by inelastic collisions with the surrounding gas, see e.g., [48]. In that case, the midplane ionization might be dominated by the decay of ^26^Al, resulting in a much lower midplane ionization rate of 4 × 10^−19^s^−1^ [49]. The results of this paper are still valid then, but with the time-axis re-scaled. If the ionization rate is indeed as low as 4 × 10^−19^s^−1^, there will be indeed no further chemical activity in the midplane during the lifetime of the protoplanetary disk. If, however, the ionization rate is 10× or 100× larger, for example if the star formation region has produced nearby supernova explosions, which produce new cosmic rays locally, the conversion to C*/*O → 1 might take only several 10^5^ or 10^4^ years, respectively.

The large initial quantities of O_2_ predicted by our disk model may not be a reliable result. Molecular oxygen remains undetected in a small number of observed interstellar clouds of varying ages [50,51]. These observational findings by the SWAS/Odin missions have triggered new world-wide efforts to refine the chemical rate networks, and to include additional complicated surface chemical processes. Different groups have presented different solutions how to keep the O_2_ concentration within the observed limits under dark cloud conditions [44,45]. Assuming quite young ages for the clouds seems to provide the easiest solution [39], but typically results in an overabundance of gaseous H_2_O [52]. We, too, can avoid the over-prediction of O_2_ in the dark cloud model by assuming low densities and young cloud ages, but we cannot avoid the formation of massive amounts of O_2_ in the disk model during the first ∼10^5^ years under the high-density conditions in the disk, neither with the UMIST-2012, nor with the OSU-2010 reaction rates. We are possibly missing some complicated surface-chemical processes which convert O_2_ on contact with dust grain surfaces.

An alternative idea how to resolve the O_2_ mystery is to consider discharge processes (lightning) like in substellar atmospheres. The degree of ionization of the disk midplane can be altered, at least temporarily, by small-scale or large-scale discharge processes between single grains or ensembles of grains that undergo collisional ionization due to the turbulent character of the midplane disk material [53,54,55]. Such processes might trigger Alfvén ionization of the O_2_ and other molecules in a weakly magnetized disk [56] potentially increasing the chemical activity in the disk midplane.

Another effect, that has been disregarded in this paper, is that large planetesimals, especially the large parental bodies that form during oligarchic growth, may heat up internally due to the heating provided by radioactive decay of ^26^Al [57]. The question arises then to what degree planet cores lose their ices, or even the more volatile constituents of their refractory material, just like comets do when they come too close to the sun, prior to the run-away gas accretion. These questions, however, go beyond the scope of this paper. If the ice elements C, N and O are completely returned to the gas phase, just prior to the rapid phase of gas accretion, the gas might re-gain its primordial C/N/O element composition, or may even locally exceed it, if the dust/gas ratio was locally enhanced, for example due to gravitational settling.

## 3. Cloud Formation Processes in Extrasolar Planets

Inspired by the complexity of the changing carbon and oxygen abundances during the evolution of a protoplanetary disk, we present first tests of the impact of changing oxygen and carbon abundances on the cloud formation in ultra-cool, planetary atmospheres as one of the essential modeling complexes.

All solar system planets with an atmospheres have clouds of various compositions. Only our Earth has exactly the right amount of clouds to allow vegetation to grow by letting the Sun-light pass through while still protecting the surface from too much Sun-light, and by transporting and releasing water over the continents. Extrasolar planets should also have clouds because their atmospheres are sufficiently cool, but their composition will be very different from what we know from Earth and the solar system; they are made of various kinds of minerals in giant gas planets or in warm atmosphere portions of cooler planets [2,3]. Transit observations of the exo-planet HD189733b [58,59] support these results by suggesting the presence of small silicate grains (haze) in the upper layers. Sub-micron size aerosol particles were suggested in the upper atmosphere of WASP-12b [60]. To complicate things, we cannot take cloud measurements of these exoplanets like we do for Earth and, to some extant, for Venus and Jupiter. This situation requires us to think carefully about the cloud formation processes and their consistent modeling in much more detail than needed for the solar system. The emphasis is here on the coupling and simultaneous treatment of all possible processes as local situations can vastly change from exo-planet to exo-planet. We will summaries the processes that lead to and are involved in the formation of atmospheric clouds in ultra-cool, planetary objects in the next section, and discuss relevant results of our cloud formation model for illustration. A comprehensive outline of the body of equations of our kinetic approach to cloud formation is given in [61].

*Approach used:* All following results were obtained by solving a set of moment and element conservation equations (see [2,62,63]) for a prescribed model atmosphere structure. The moment equations describe the seed formation, growth/evaporation and gravitational settling for mixed grains made of 12 material species that form by 60 surface reactions. We use a Drift-Phoenix model structure for gas temperature, gas pressure and convective velocity as input [3]. Drift-Phoenix models use the same set of cloud model equations, only the number of condensing materials in lower than considered in this paper. The initial values for the element abundances were solar [1] but do change due to cloud formation as shown in Figure 5. We will depart from this assumption in Section 4. We note that the Drift-Phoenix model structure are calculated without the effect of an external radiation source.

### 3.1. Cloud Formation Processes

Cloud formation is an intrinsic non-equilibrium process during which the gas-phase constituents participate in a phase-transition from which the cloud particles emerge. No cloud particle can *form* in phase-equilibrium as the very nature of an equilibrium is to be the minimum energy state of a system where the system feels very comfortable in.

**Figure 4:**
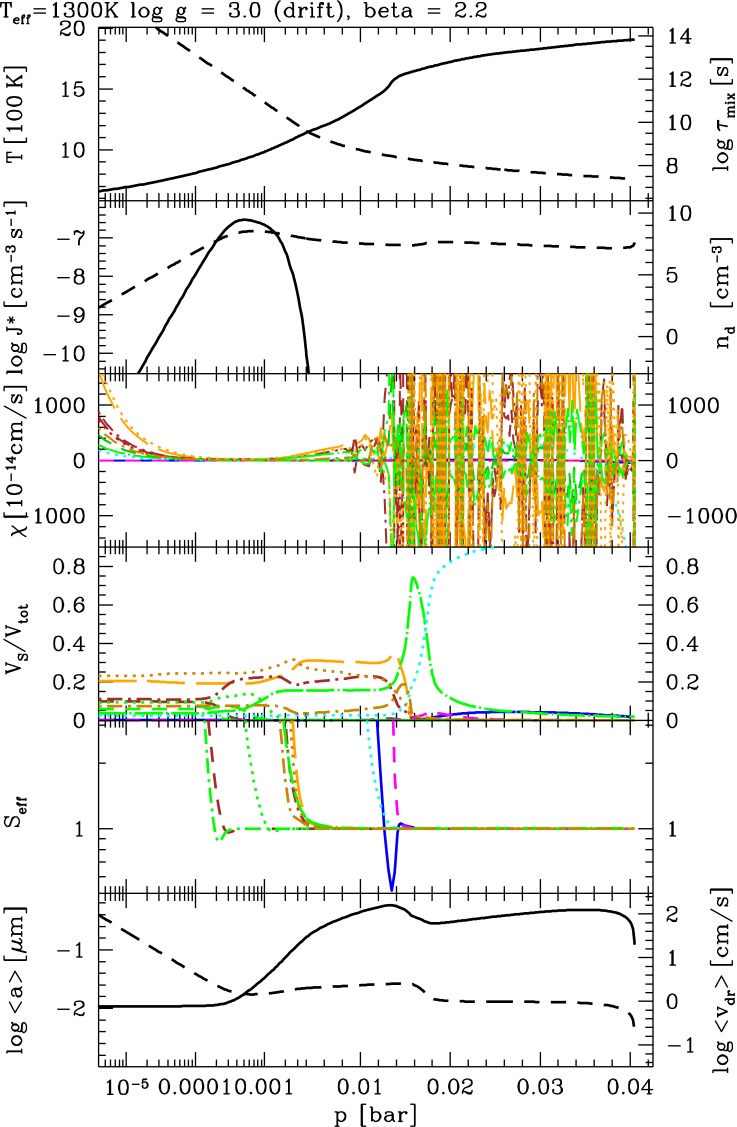
Structure and physical properties of a mineral cloud in the atmosphere of a planet with T_eff_ = 1300 K, log(g) = 3.0 and solar element abundances. The model atmosphere structure is taken from the Drift-Phoenix grid [3]. The solar Drift-Phoenix grid spans T_eff_ = 1000. . . 3000 K, log(g) = 3.0. . . 6.0. **1st panel:** left—gas phase temperature T_gas_ [K], right—time scale of convective up-mixing *τ*_mix_ [s]; **2nd panel:** left—nucleation rate J_*_ [cm^−3^ s^−1^], right—number density of dust particles n_d_ [cm^−3^]; **3rd panel:** growth velocity of different materials χ [cm/s]; **4th panel:** particle material composition in volume fraction V/V_s_ (∑_s_ *V*_s_—total dust volume); **5th panel:** effective supersaturation ratio for each material S_eff_; **6th panel:** left—cloud particle mean size *<*a*>* [μm], right—mean drift velocity v_dr_ [cm/s]. The color/line coding is the same for all panels and plots: TiO_2_[s]—solid blue, Mg_2_SiO_4_[s]—orange long-dash, MgO[s]—dark orange dot dash, SiO[s]—brown dost short dash, SiO_2_[s]—brown dot dash; Fe[s]—green dot long dash ; Al_2_O_3_[s]—cyan dotted, CaTiO3_3_[s]—magenta dashed. Only a subset of all 12 materials is depicted.

Cloud formation in extrasolar atmospheres necessarily starts with the *formation of seed particles* because we cannot assume that the planetary object has a crust from which sand or ash particles are diffused upwards or injected into the atmosphere by volcanic eruptions. In terrestrial atmospheric literature, *seed particles* are referred to as *cloud condensation nuclei*. These are particles able to promote droplet formation at terrestrial atmospheric water supersaturation levels. In more general terms, such seeds provide a surface onto which other material can condense more easily as surface reactions are considerably more efficient than the sum of chemical reactions leading to the formation of the seed. Figure 4 summarizes the results of our cloud model for one example atmosphere model.

*Formation of seed particles:* The formation of the first surface out of the gas phase proceeds by a number of subsequent chemical reactions that eventually result in small seed particles. Such a chain of chemical reactions can proceed by adding the same molecular unit (=monomer) during each reaction step (e.g., [64]) which is referred to as *homogeneous nucleation. Heterogeneous nucleation* occurs if different monomer units participate in different reaction steps to form larger molecules and eventually clusters (e.g., [65,66]). We apply the concept of homogeneous nucleation to the formation of TiO_2_ seed particles. Figure 4 (2nd panel, left) shows that the nucleation rate (J_*_) peaks rather high in the atmosphere and falls off towards higher gas temperature (1st panel, left). The peak of the cloud particle number density (n_d_) coincides with the peak of the nucleation rate but the number density remains high towards higher temperature. This is a clear sign that cloud particles fall into the atmosphere and therefore do exist below the seed formation region.

*Growth, evaporation:* Growth and evaporation are surface reaction onto a surface or off a surface, respectively. They are determined by the composition of the gas phase that provides the number density for surface reactions and leads to the formation of a substantial mantle of a grain or droplet on top of the seed. This mantle determines the mass, volume and main chemical composition of the cloud particles. Many materials can be simultaneously thermally stable in a gas but these materials change depending on the carbon-to-oxygen ratio and the abundance ratios of other elements. In principle, all stable and supersaturated material can grow simultaneously on a seed particle. Figure 4 (5th panel) shows the effective supersaturation ratios (S_eff_, [2]) for all materials involved here (TiO_2_[s], SiO[s], SiO_2_[s], Fe[s], FeO[s], Fe_2_O_3_[s], FeS[s], MgO[s], MgSiO_3_[s], Mg_2_SiO_4_[s], Al_2_O_3_[s], CaTiO_3_[s] with the corresponding surface reactions as in Table 1 in [2]). This demonstrates that all materials are supersaturated at high atmospheric layers and subsequently achieve phase equilibrium deeper in the cloud: They grow until the gas-phase has reached phase-equilibrium for these particular materials. High-temperature condensates like Al_2_O_3_[s] (light blue), TiO_2_[s] (dark blue) and CaTiO_3_[s] (magenta) reach S = 1 at considerably higher temperatures where they under-saturated eventually and evaporate. This is particularly interesting if solid particles form from the gas-phase as the composing materials comprise silicates, oxides, and iron compounds leading to the formation of grains of mixed materials [2,3,61,67]. The material composition is shown in 4th panel in Figure 4 which only depicts silicates in orange/brown colors, Fe[s] and Fe-compounds in green, Al_2_O_3_[s] in light blue, TiO_2_[s] in dark blue, and CaTiO_3_[s] in magenta.

*Gravitational settling (rain-out)*: The equilibrium between friction and gravity determine how fast the cloud particles fall through the atmosphere ([62]). The cloud particles will continue to grow and to change their material composition during their way into denser and warmer atmospheric layers (Figure 4, 4th panel). Panel 2 of the same figure shows that the cloud expands well blow the region of efficient nucleation, hence, these particle fall in from above. The mean grain size (6th panel) is determined by the different dust formation processes that govern the dust formation at different sites in the atmosphere: The upper part of the cloud is governed by the nucleation process. Growth is very inefficient due to the low density of the ambient gas, hence, the cloud particles remain very small and haze-like. Once the particles sink into deeper layers, the surface growth process dominates, resulting in strongly increasing grain sizes, until *p*_gas_ ∼ 0.001 bar in the model depicted. It follows a small minimum where all the silicates evaporate (compare 4th panel). Fe[s]-growth picks up but the grain size does not change considerably during their remaining path through the atmosphere. A comparison with the drift velocity (6th panel, right) shows that the grains fall with almost constant speed through the atmosphere until they evaporate at the bottom of the cloud.

**Figure 5:**
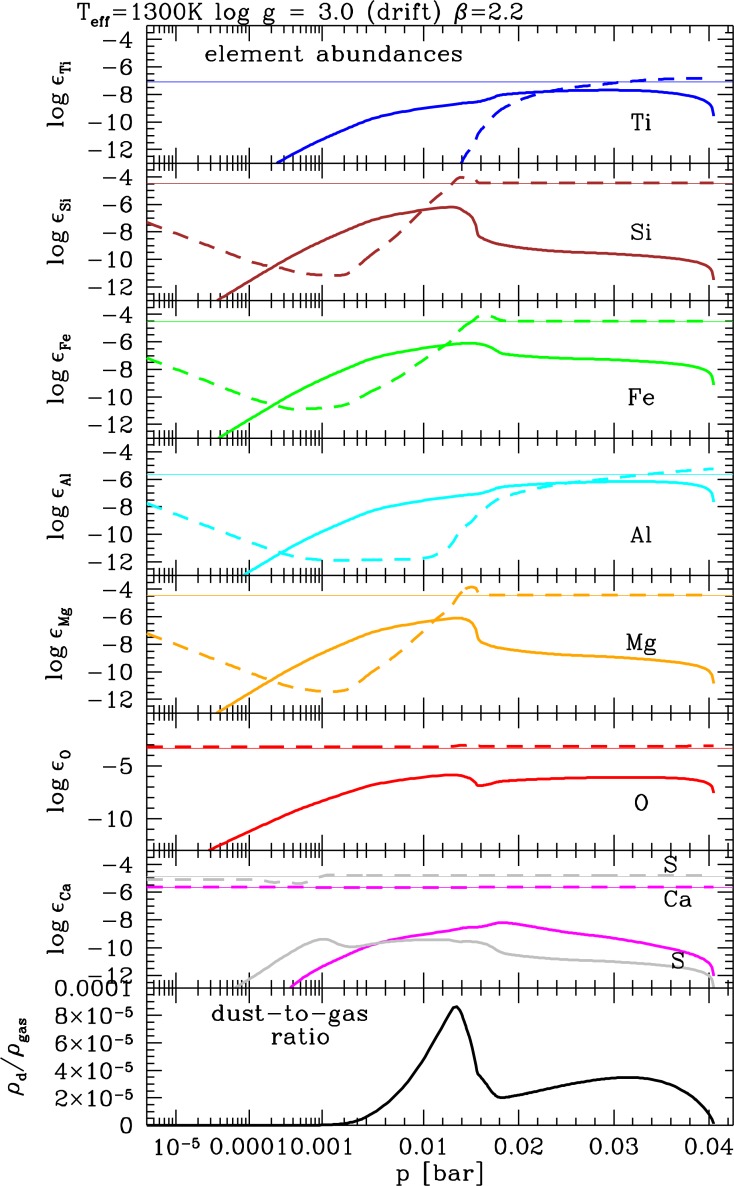
Element abundances changing through mineral cloud formation in the atmosphere of a planet for the same model like in Figure 4. Plotted are the initial solar abundances (thin solid line), the actual gas-phase element abundances (dashed line), and the element abundances locked into the dust (thick solid line). The dust-to-gas ratio, *ρ*_d_/*ρ*_g_, is depicted for comparison (lowest panel).

*Element depletion due to cloud formation:* We have summarized the cloud formation processes above. The cloud formation has a strong impact on the local chemistry by depleting those elements that participate in the formation of the cloud particles. Figure 5 demonstrates how the gas-phase element abundances change compared to the initial, canonical solar value for all elements that participate in the cloud particle formation in our model. The comparison with the dust-to-gas ratio (*ρ*_d_*/ρ*_g_, lowest panel) shows that most of the dust is present when most of the elements Mg, Si and O are locked in dust. Figure 5 also emphasises that a C/O ratio alone is not sufficient to characterise the element abundances of an objects as elements change their abundances individually according to their involvement in cloud formation (or ice condensation in the protoplanetary disk to start with).

## 4. Changing Cloud Properties in Atmospheres with Non-solar Abundances

Cloud formation is determined by the local atmospheric properties, T_gas_ and *ρ*_gas_, and the local element abundances, *∊*_i_. Cloud formation has a strong feedback on *∊*_i_ due to element-dependent lock-up in the cloud particles, and on T_gas_ due to the dust opacity and due to element depletion that changes the gas opacity. Studying cloud formation in atmospheres with non-solar abundances affords us a first assessment of how different the local chemistry might be in newly forming planets that are exposed to changing element abundances with distance from the star and during the history of disk evolution itself. It is of importance to understand to which extent element abundances influence the results of our cloud modeling and/or the local gas-phase chemistry, and how this might require a re-interpretation of, for example, the tentative detection of carbon planets or the conclusions draw from accumulating uncertainties in element depletion by phase-equilibrium cloud models, initial element abundances, mixing ratios and quenching heights [4,31,32]. We concentrate on the effect of element abundances only and do not consider irradiation, photo- or ion-chemistry, nor atmospheric circulation. Our results will therefore be directly applicable to planets in the outer portion of the disk where the host star’s radiation field does not play a significant role. Here, the chemical composition in upper atmosphere above the cloud layer will be influences by cosmic ray chemistry [68]. Atmosphere models that take into account the presence of clouds in irradiated planets [69] consider a host star distance of *<*0.05 AU which is in the oxygen-rich part of our disk models where oxygen-reducing ice formation does not play a role (Figure 1 and Figure 2). The following study is applicable to directly imaged planets [30,70,71,72,73,74,75] and free-floating planets, e.g., [76,77]. The issue of non-solar element abundances is also relevant for the large number of close-in, irradiated planets as the element abundances are an essential input *and* output quantity.

### 4.1. Sub- and Super-solar Oxygen and Carbon Abundances

After presenting the canonical result of our cloud formation model for a planetary atmosphere with the solar element abundances as initial values (Section 3), we manipulate the initial values for the oxygen and carbon abundances guided by the ProDiMo disk model results (Section 2). Our aim is to present a first study of how much the cloud formation processes in a planetary atmosphere would change for different C/O ratios as to be expected in a protoplanetary disk. As pointed out in Section 2.3, all other elements can change too, but oxygen and carbon will dominate the remaining disk chemistry. Figure 1 and Figure 2 suggest that the C/O ratio changes with disk age and with increasing radial distance from the star. The reason is the formation of water ice (H_2_O#) and CO-ice (CO#) at different radial distances *>*0.5 AU (*ice lines*). An almost pure H_2_/He gas is left already in young disks at radial distances beyond the CO_2_-ice line, hence, young planets forming at such distances would have an extremely metal-poor atmosphere in contrast to their host star’s element composition. At smaller radial distances from the star *<*13 AU, the oxygen and carbon abundances change time-dependently in the sandwich zone between the H_2_O-ice and the CO_2_-ice lines.

In order to understand which effects changing C/O ratios have on the cloud structure, we consider lower (log *∊*_O_ = 8.6, log *∊*_C_ = 8.4) and higher (log *∊*_O_ = 8.9, log *∊*_C_ = 8.8) oxygen and carbon abundances compared to the solar values (log ${∊}_{\text{O}}^{\text{solar}}$ = 8.87, log ${∊}_{\text{C}}^{\text{solar}}$ = 8.55) that are commonly used in model atmosphere simulations. All other input quantities are kept the same. Element abundances are given relative to the hydrogen abundance (log *∊*_H_ = 12).

**Figure 6:**
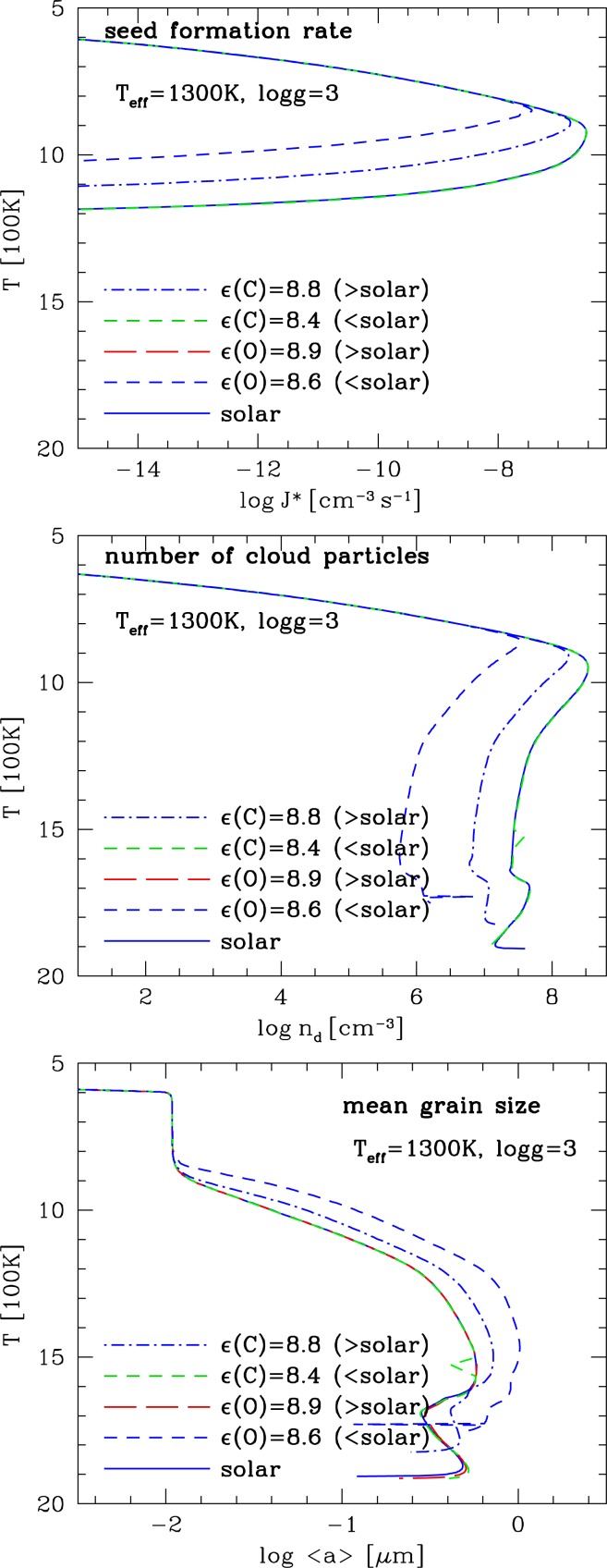
Cloud properties for different oxygen (*∊* (O)) or carbon *∊* (C)) abundances for a pre-scribed Drift-Phoenix planetary model atmospheres (T_eff_ =1300K, log(g)=3.0). **Top:** nucleation rate, J_*_ [cm^−3^ s^−1^]; **Middle:** number of cloud particles, n_d_ [cm^−3^]; **Bottom:** mean size of cloud particles, *<*a*>* [μm].

Figure 6 shows that fundamental cloud properties change due only to a change in oxygen/carbon abundance: The seed formation rate (top panel, TiO_2_ seeds) decreases with decreasing oxygen abundance and increasing carbon abundance, and this has fundamental implications for the cloud opacity as it changes the mean grain size (bottom panel). Decreasing oxygen and increasing carbon abundance have the same net effect of decreasing the amount of oxygen available for TiO_2_ molecules (seed monomer) to be formed. If more carbon is available, more oxygen will be locked in carbon-monoxide (CO). Hence, already small changes in *∊*_O_ and *∊*_C_ affect the Ti-chemistry because Ti has a very low element abundance (see [63] Figure 5).

The lower rate of seed formation results in less cloud particles being formed (middle panel, Figure 6). Clouds in a low-oxygen abundance gas (but still C/O*<*1; dashed and long-dashed line) do have fewer cloud particles, suggesting a more transparent haze layer. These particles will rain into the denser atmosphere and grow to bigger sizes than in the solar abundance case (solid line). The maximum grain size increases from 0.4μm in the solar case to about 1μm due solely to a moderate decrease of the oxygen abundance (or increase of *∊*_C_). We note that an increase of *∊*_O_ to values larger than the solar values and a decrease of *∊*_C_ below the solar value does not have any effect on the cloud properties. The reason is that all possible binding partners (Si, Fe, Mg, Al) to O have much lower element abundances and are already locked up in molecules. The CO molecule acts as an oxygen-sink and no increase of carbon could change this under the conditions of gas-phase chemical equilibrium applied here. This leads to the conclusion that the cloud properties would change further if the Si, Fe, Mg, Al abundances would change too. At a first glance, this would mean that less material could grow onto seed particles. However, the gas-phase chemistry may offer other surface reactions than those used in our cloud formation model (Table 1, [2]).

**Figure 7:**
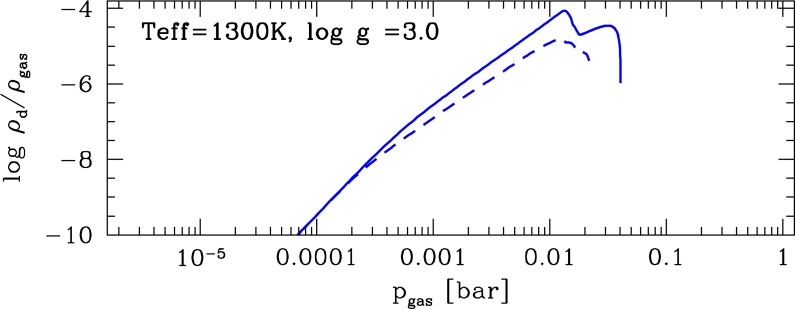
Changing atmospheric dust-to-gas ratio with changing initial element abundances for models depicted in Figure 6. Shown are the solar case (solid line) and the sub-solar case for *∊*_O_ = 8.6 (dashed line). No differences in the *ρ*_d_*/ρ*_g_ ratio were found for the other cases.

We test the implication of the changing effective oxygen abundance on the dust-to-gas ratio, *ρ*_d_*/ρ*_g_, in planetary atmospheres (Figure 7). The largest change of about a factor of 10 occurs for a decreasing oxygen abundance which supports our conclusions from Figure 1 and Figure 2 above.

### 4.2. Sub-solar Oxygen and Carbon Abundances at the Extreme: C/O = 0.99

**Figure 8:**
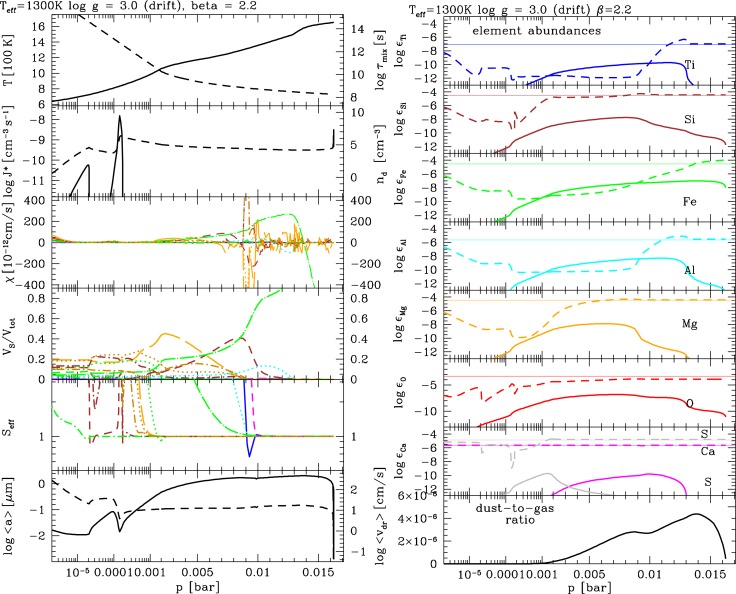
Cloud structure results for a gas of C/O_init_=0.99 *∊*_O_ = 8.07, *∊*_C_ = 8.06) for the same Drift-Phoenix atmosphere model as in the previous figures. **Left:** Cloud structure and physical properties. **Right:** Element abundances changing through mineral cloud formation. In both figures, the same line coding is used as in Figure 4 and Figure 5.

The outstanding question is if carbon-rich planets could exist around oxygen-rich host stars. The core-accretion scenario for planet formation does not presently suggest the in-situ formation of carbon-rich planets, unless models are very much simplified to match the observations. We have shown that cloud formation might play a crucial role in tipping over an oxygen-rich atmospheric gas composition locally towards a carbon-rich atmospheric gas, due to the additional formation of oxygen-rich dust in the atmosphere. We have tested this hypothesis by decreasing the oxygen abundances in our cloud formation Drift code to values as low as suggested by ProDiMo (*∊*_O_ = 8.07, *∊*_C_ = 8.06) approaching C*/*O ≈ 1. Figure 8 shows the results for the cloud structure (left) and the element abundances (right): The seed formation rate is very low compared to all results from the previous sections due to the much lower oxygen abundance. TiO_2_-seed formation is still possible due to a small surplus of oxygen that is not locked by CO. But considerably less particles are formed and two detached nucleation maxima determine the number of cloud particles. The upper most nucleation event is killed off by the oxygen-consumption during the growth process involving TiO-molecules. Nucleation resumes only where the atmospheric density is sufficiently high and the temperature sufficiently low. Note that Figure 8 (left) shows the peak values of the nucleation rate, and that the atmospheric range affected by nucleation and growth extends towards considerably lower pressures (compare Figure 8, right).

The competition for condensable material become apparent from the double-peaked nucleation rate J_*_: The growth of silicates consumed oxygen which decreases the number of TiO_2_ molecules in the gas, causing the nucleation process to stop. A local increase of the mean grain size results and the grains form a semi-detached haze layer of 0.1 μm silicate grains. The second nucleation peak coincides with a decreasing grain size just on top of a deeper cloud layer of an almost constant grain size of 1 μm.

### 4.3. Implications for the Chemical Composition of the Atmosphere

**Figure 9:**
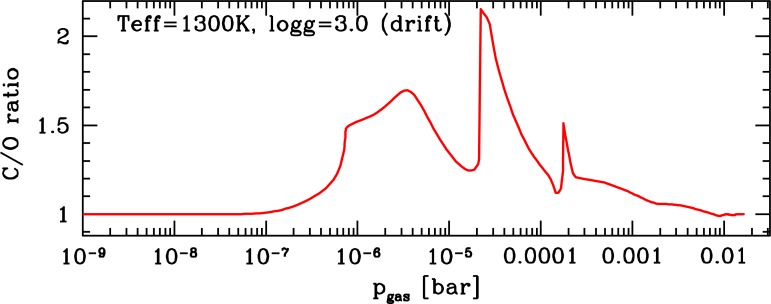
C/O ratio after cloud formation from an initial element abundance with C/O_init_ = 0.99 (*∊*_O_ = 8.07, *∊*_C_ = 8.06; all other elements solar). The oxygen depletion by cloud formation clearly tips the gas-phase from an oxygen-dominated to a carbon-dominated chemistry.

The gas-phase composition is determined by the local temperature (and density) and the local element abundances. Both are affected by cloud formation since cloud formation causes a considerable element depletion of the gas phase. The C/O-ratio of a cloud forming atmosphere with an initial C/O_init_ = 0.99 can increase to values C/O*>* 1 . . . 2 alone due to oxygen depletion by cloud formation (Figure 9). As a result of cloud formation, the atmosphere can become locally very carbon-rich. We, however, do not find C/O ratios ∼100. Our results suggest further that young planets that accrete their first atmosphere from the disk dust and gas will most likely have a strongly oxygen-depleted. This is because the primordial gas will condense more easily onto the previous disk grains due to an increased local density during accretion. Cosmic Rays can enhance the fraction of hydro-carbon molecules (via ion-neutral reaction) [68], a process that can be considerably more efficient in an atmosphere that is oxygen-depleted as result of disk evolution and cloud formation.

**Figure 10:**
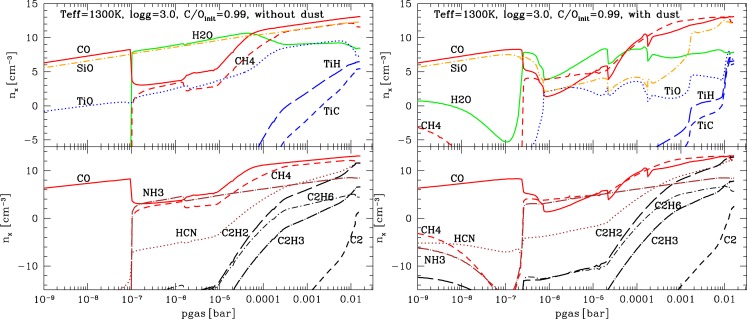
Atmospheric molecular number densities in chemical equilibrium for the planetary model atmosphere in Figure 8. **Left:** No element depletion by dust formation (C/O_init_ = 0.99), **Right:** With element depletion by dust formation (O, Si, Mg, Fe, Ti, Ca, Al) resulting in the C/O ratio depicted in Figure 9 (C/O *>* 1 . . . 2).

We have performed a calculation of the gas-phase composition of the atmosphere depicted in Figure 8 by applying our chemical equilibrium code [4]. This code allows us to see how the abundance of molecules that are strong opacity carriers in oxygen-rich atmosphere (H_2_O, CO, SiO, TiO) change if the local C/O ratio changes. We also consider CH_4_ and NH_3_ which are discussed as possible bio-marker molecules, and hydrocarbon chains and cyano-molecules. The abundance of these molecules is shown in the left panel of Figure 10 for C/O_init_ = 0.99 with no element depletion by dust formation as reference values for the case with element depletion by dust formation (right pane) causing C/O*>* 1. In the left panel, the dust only influences the local temperature structure causing the drop of CO at 10^−7^ bar in the model results shown. We note the increasing abundance of hydrocarbon chains (C_2_H_2_, C_2_H_6_, C_2_H_3_) and HCN with increasing pressure below the cloud layer. The molecular chemical equilibrium abundances for a gas with C/O close to unity (but not quit 1) is very sensitive to small changes in the oxygen/carbon abundance. This result is not new and was discussed for S-type AGB stars by e.g., [78]. Hydrocarbon absorption were observed in S-type AGB stars while it is surprisingly difficult to identify PAH absorption feature in carbon-rich AGB stars, e.g., [79].

The number densities of all typical oxygen-rich gas phase molecules decreases considerably with C/O*>* 1. In the cloud layers, H_2_O remains the most abundant species after H_2_. The H_2_O, CO and the SiO gas abundances in particular are a negative fingerprint of the dust growth process (e.g., 4th panel, left of Figure 8). CH_4_ and NH_3_ become more abundant than CO in the cloud formation zone because it is not affected by the element depletion due to cloud formation. As before, NH_3_ and CH_4_ are of similar abundance, but CH_4_ is somewhat more abundant than NH_3_. The largest difference between the depleted (right of Figure 10) and the un-depleted case (left of Figure 10) is the increasing abundances of H_2_O, CH_4_, HCN, NH_3_, C_2_H_2_ and C_2_H_6_ in the low-pressure regime near the cloud top. HCN becomes relatively more important which is typical for low-metallicity gases near C/O = 1.

## 5. Conclusions

We have discussed the chemical preconditions for planet formation in protoplanetary disks, with emphasis on the time-dependent segregation of carbon, nitrogen and oxygen into gas and ice phases. We obtained the following results:

- The segregation into gas and ice phases beyond the water ice-line (the “snowline”) result in a rich variety of gaseous oxygen, carbon, and nitrogen abundances in the midplanes of protoplanetary disks, depending on time, position in the disk, and cosmic ray ionization rate. The resulting gas element abundances can vastly differ from that of the host star.
- Inside of the snowline (≳150 K) all considered ice phases are thermally unstable, and the gas phase abundances remain primordial.
- Beyond the CO ice-line (≲20 K) oxygen, carbon and nitrogen freeze out quickly, and already after ≪10^3^ years, the outer midplane barely contains any molecules other than H_2_. This may be different though, for the outermost midplane which is transparent to interstellar UV and X-ray irradiation, as well as for scattered stellar UV and X-ray irradiation.
- Between the snowline and the CO ice-line, a slow transition from O-rich to C*/*O → 1 takes place, on timescales of ∼ 3 Myrs. This timescale is related to the cosmic-ray induced un-blocking of O_2_ and CO, and scales with the cosmic ray ionization rate assumed.
- For very long-lived protoplanetary disks, or disks exposed to an unusually high cosmic ray ionization rate, the carbon-to-oxygen ratio C/O would eventually exceed unity, leading to a sudden occurrence of organic molecules in the midplane, and providing the chemical pre-conditions for the formation of carbon planets.

Following the standard core-accretion model, it is this element-depleted gas that will be finally accreted onto the proto-planet in a rapid run-away phase, to eventually form the planetary atmosphere, although many difficult questions remain open, like the subsequent bombardment with left-over planetesimals, or the previous internal heating and outgasing of the planetesimals due to radioactive decay of ^26^Al. However, in agreement with [17], we conclude that a super-solar C/O ratio (but C*/*O≲1) is the most likely result from the gas-ice segregation in the disk, between the snowline and the CO ice-line.

In the remainder of this paper, we have studied how the cloud formation in young planetary atmospheres is affected by decreased oxygen abundances. Our results are applicable to directly imaged planets like HB 8799b,c,d,e, GQ Lupi or *β* Pic b, and free-floating planets. But the issue of non-solar element abundances has biased also our understanding of the atmospheres of the large number of close-in, irradiated planets. Cloud formation depends strongly on the local element abundances involved (Fe, Ti, Si, O, . . .) and it strongly affects the local elements by element depletion or enrichment. The consequence is a strong influence of the cloud formation on the local opacity, and hence on the atmosphere’s temperature structure. The oxygen abundance has a strong impact on the seed particle formation rate which initiates the cloud formation process. The reduced number of seed particles leads to a lower number density of cloud particles which hence grow to larger sizes throughout most of the cloud in an atmosphere with C*/*O≲1. This sequence of processes leads to a more transparent haze layer on low-C/O planet compared to a solar abundance (or solar-abdundance scaled) atmospheres. However, an increasing oxygen abundance does not automatically cause more cloud particles to be formed because the nucleation rate is determined by the monomer density, not by the oxygen abundance alone. We further observe the appearance of a semi-detached cloud layer with C/O→1.

We conclude that the differences in element abundances with radial distance in protoplanetary disk have broad implications for the cloud properties in planetary atmospheres. The element abundances are, however not a multiple of the set of solar abundances. Using the results of our disk models as input for our cloud and chemistry calculation did only result in C/O ≈ 2, and and we can not confirm element abundances in planetary atmospheres as high as 100× the solar values. Planetary atmospheres might still carry signatures of the initial abundances of the gas in the protoplanetary disks from which they were once formed. However, it seems difficult to detect these signatures from spectroscopy directly, because cloud formation changes the gaseous element abundances.

We further have demonstrated that it is not straight-forward to argue for the formation of carbon-rich planets (Figure 1 and Figure 2) and that some fine-tuning would be necessary in order to end up with a planet that exhibits spectral signatures typical for a carbon-rich gas. So far, only additional processes like element depletion by dust cloud formation and condensation adequately explain observations of planetary atmospheres rich in carbon-binding molecules.
